# Three-Dimensional Vibration Model of Cylindrical Shells via Carrera Unified Formulation

**DOI:** 10.3390/ma16093345

**Published:** 2023-04-24

**Authors:** Weige Liang, Tao Liu, Chi Li, Qingshan Wang

**Affiliations:** 1 College of Weapons Engineering, Naval University of Engineering, Wuhan 430033, China; 1312021010@nue.edu.cn (W.L.);; 2 Light Alloy Research Institute, Central South University, Changsha 410083, China; 3 State Key Laboratory of Precision Manufacturing for Extreme Service Performance, Central South University, Changsha 410083, China

**Keywords:** three-dimensional vibration, cylindrical shell structures, unified solution, two-dimensional Chebyshev polynomials, new numerical model, elastic boundary conditions

## Abstract

In this paper, we present a novel and unified model for studying the vibration of cylindrical shells based on the three-dimensional (3D) elastic theory and the Carrera Unified Formulation. Our approach represents a significant advancement in the field, as it enables us to accurately predict the vibrational behavior of cylindrical shells under arbitrary boundary conditions. To accomplish this, we expand the axial, circumferential, and radial displacements of the shell using Chebyshev polynomials and Taylor series, thereby reducing the dimensionality of the expansion and ensuring the precision and rigor of our results. In addition, we introduce three groups of artificial boundary surface springs to simulate the general end boundary conditions of the cylindrical shell and coupling springs to strongly couple the two surfaces of the cylindrical shell *φ* = 0 and *φ* = 2π to ensure continuity of displacements on these faces. Using the energy function of the entire cylindrical shell model, we obtain the characteristic equation of the system by finding the partial derivatives of the unknown coefficients of displacement in the energy function. By solving this equation, we can directly obtain the vibration characteristics of the cylindrical shell. We demonstrate the convergence, accuracy, and reliability of our approach by comparing our computational results with existing results in the literature and finite element results. Finally, we present simulation results of the frequency features of cylindrical shells with various geometrical and boundary parameters in the form of tables and figures. Overall, we believe that our novel approach has the potential to greatly enhance our understanding of cylindrical shells and pave the way for further advancements in the field of structural engineering. Our comprehensive model and simulation results contribute to the ongoing efforts to develop efficient and reliable techniques for analyzing the vibrational behavior of cylindrical shells.

## 1. Introduction

The cylindrical shell is one of the most significant and commonly used structural elements in a variety of military and civilian applications. However, due to various complex loads and environmental influences during the working process of cylindrical shell structures, they are prone to phenomena such as vibration and sound radiation. This not only affects their normal function and service life but also poses a threat to personnel safety and environmental protection. Therefore, studying the vibration characteristics of cylindrical shell structures has important theoretical significance and practical value for aspects such as optimizing design, controlling noise, improving reliability, and extending service life.

**Figure 1:**
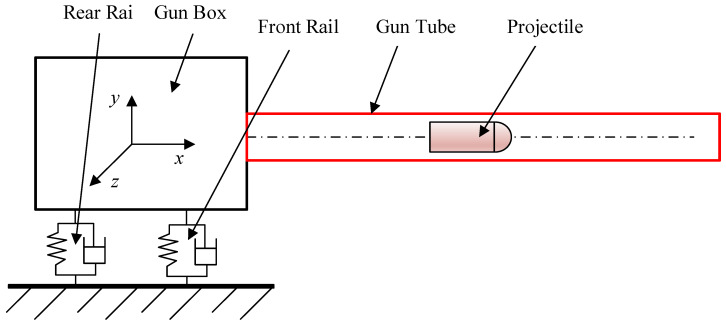
The physical model of an automatic gun.

As shown in Figure 1, it is a simplified artillery model, whose barrel is a typical cylindrical shell structure. Usually, for the study of the dynamics of the gun barrel, the beam theory was mainly used to simplify the modeling [1,2]. Yet in fact, the theory of the beam model only takes into account the radial vibration of the barrel, which cannot describe the complete dynamics of the cylindrical barrel. Therefore, it is necessary to establish the cylindrical shell model using 3D elastic theory for analysis to obtain accurate vibration characteristics of the cylindrical shell.

There is a lot of literature on the 3D vibration studies of cylindrical shells. Liew and Hung [3] used the orthogonal polynomial functions to approximate the displacement field of a solid cylinder and obtained the 3D vibration characteristics of the solid cylinder. Loy and Lam [4] used a layered approach to study the vibration of thick cylindrical shells with simple-support–simple-support and clamped–clamped boundaries. The cylindrical shell was discretized in the thickness direction into an arbitrary number of thin cylindrical layers, and the displacement of each layer was approximated by trigonometric functions in the axial and circumferential directions, as well as some linear shape functions in the thickness direction. Zhou et al. [5] took the Chebyshev polynomials as the admissible functions for cylindrical shells, and then adopted the Ritz method to obtain the vibrations of cylindrical shells. Mofakhami et al. [6] published a general solution to examine the vibration of cylinders with various boundary conditions by using an infinite cylinder solution based on the variable separation approach. Khalili et al. [7] presented the free vibration analysis of isotropic circular cylindrical shells by using the 3D refined higher-order shear deformation theory. On the dynamic response of cylindrical shells, the theory considered the effects of in-plane and rotational inertia, as well as transverse normal and shear strains. Because the stresses over the shell thickness must be integrated over the trapezoidal cross section of the shell elements to obtain accurate stress results, the trapezoidal shape factor of the shell elements was also included. By combining the layered theory with the 3D form of the Hamiltonian principle and the associated boundary conditions, Malekzadeh et al. [8] obtained the equations of motion for a 3D cylindrical shell, and the obtained equations were discretized using the differential product method. Ye et al. [9] proposed a unified solution for the free vibration of thick cylindrical shells, the shell displacements were expanded as a standard Fourier series with auxiliary functions, and the energy functions of the shell were solved by the Rayleigh—Ritz method. Atashipour et al. [10] presented a novel 3D elastic motion including the body force in a cylindrical coordinate system; the 3D static and free vibrations of a hollow cylindrical shell were solved analytically.

At present, there are many different theories applied to the study of vibration of cylindrical shell structures in addition to the three-dimensional theory, which are listed in Table 1. As can be seen from the table, researchers have developed different methods based on different theories to analyze the vibration of cylindrical shell structures of different materials and have obtained a large amount of valuable data.

Recently, there are also some studies on the 3D vibration of composite cylindrical shell structures. Su et al. [21] analyzed the 3D vibration of functionally graded conical and cylindrical shells by using the Modified Fourier series method. Qin et al. [22] used the 3D elastic theory and Rayleigh—Ritz method to analyze the free vibration of functionally graded porous graphene platelet-reinforced composite cylindrical shells. They compared six different types of admissible functions including Chebyshev polynomials of the first kind, Chebyshev polynomials of the second kind, Legendre polynomials, Orthogonal polynomials, Modified Fourier series of the first kind, and Modified Fourier series of the second kind. Thanh et al. [23] presented a 3D numerical solution based on isogeometric analysis to investigate the free vibration of cylindrical shells made of functionally graded porous-cellular materials. Tong et al. [24] proposed a solution for free vibration analysis of arbitrary angle-play laminated cylindrical shells, which combined the state—space technique and differential quadrature method.

The present solution model is established based on the Carrera Unified Formulation (CUF). The CUF was refined by Carrera et al. [25,26,27] after extensive research of typical structures such as beams, plates, and shells. The classical theories such as Kirchhoff and Mindlin shear deformation theories can be derived as special cases of CUF [28,29]. As a powerful general theory, CUF had been successfully applied to shell structures [30,31,32]. In CUF, the displacement fields of structures are generalized and expressed by the polynomials, such as Taylor series and Lagrange polynomials, and the precision of the CUF is primarily determined by the terms of polynomial expansions.

In the present work, a new 3D model based on CUF is proposed for free vibrations of the cylindrical shells with elastically restrained ends. The axial, circumferential and radial displacements of the cylindrical shell are expanded by Chebyshev polynomials and Taylor-like series, and the system characteristic equations are constructed by the 3D elastic shell theory. Three groups of face boundary springs are used to simulate the boundary conditions of two end surfaces of the cylindrical shell, and the two surfaces of the cylindrical shell *φ* = 0 and *φ* = 2π are strongly coupled by surface coupling springs. The vibration results of the current solution are proved by comparing them with other methods in literature and FEM software (ABAQUS 2022).

## 2. Theoretical Formulations

### 2.1. Model Description

**Figure 2:**
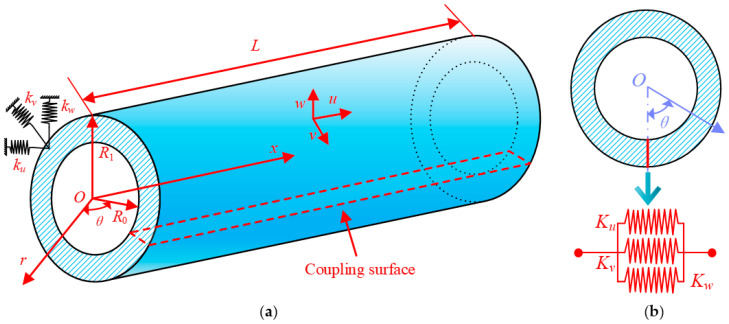
Schematic diagram of a 3D cylindrical shell: (**a**) the whole shell; (**b**) the coupling surface.

The object of interest in the paper, as depicted in Figure 2a, is a 3D cylindrical shell characterized by an interior radius of *R*_0_, an outer radius of *R*_1_, and a length of *L*. The cylindrical coordinate system is used to define the shell in this paper, and the axes *x*, *θ*, and *r* depict the axial, circumferential, and radial directions, respectively. The corresponding displacement components of any point of the shell on the three axes are given by *u*, *v*, and *w*, respectively.

The spring constraint is applied at the end of the shell to represent the boundary conditions, containing three sets of linear springs $k_{u}^{i}$, $k_{v}^{i}$, and $k_{w}^{i}$, the superscript *i* = 0 means that the boundary spring is arranged at the *x* = 0 end of the cylindrical shell, and *i* = *L* means that it is arranged at the *x* = *L* end. Thus, all homogeneous boundary conditions can be obtained directly by setting suitable spring stiffness values. In addition, the kinematic and physical continuity conditions of a complete rotating shell should be satisfied at the circumference *θ* = 0 and *θ* = 2π. Therefore, coupling springs are set in these two surfaces in this paper. The three groups of face springs are denoted by *K_u_*, *K_v_*_,_ and *K_w_* (Figure 2b).

### 2.2. Energy Expressions

In this paper, the 3D shell theory is used to establish the cylindrical model, and the strains of the shell are defined as

(1) $$\begin{array}{l} \begin{array}{ccc} \varepsilon_{x}=\frac{\partial u}{\partial x}, & \varepsilon_{\theta }=\frac{\partial v}{r\partial \theta }+\frac{w}{r}, & \varepsilon_{r}=\frac{\partial w}{\partial r} \end{array}\\ \begin{array}{ccc} \gamma_{\theta r}=\frac{\partial w}{r\partial \theta }+\frac{\partial v}{\partial r}-\frac{v}{r}, & \gamma_{xr}=\frac{\partial u}{\partial r}+\frac{\partial w}{\partial x}, & \gamma_{x\theta }=\frac{\partial u}{r\partial \theta }+\frac{\partial v}{\partial x} \end{array} \end{array}$$

where the symbols $\varepsilon_{x}$, $\varepsilon_{\theta }$, and $\varepsilon_{r}$ denote the normal strains, the symbols $\gamma_{\theta r}$, $\gamma_{xr}$, and $\gamma_{x\theta }$ are the shear strains.

The stresses can be defined by means of the generalized Hooke’s law:

(2) $$\left\{\begin{array}{c} \begin{array}{c} \begin{array}{c} \begin{array}{c} \begin{array}{c} \sigma_{x}\\ \sigma_{\theta } \end{array}\\ \sigma_{r} \end{array}\\ \tau_{\theta r} \end{array}\\ \tau_{xr} \end{array}\\ \tau_{x\theta } \end{array}\right\}=\frac{E}{\left(1+\mu \right)\left(1-2\mu \right)}\left(\begin{array}{cccccc} 1-\mu & \mu & \mu & 0 & 0 & 0\\ \mu & 1-\mu & \mu & 0 & 0 & 0\\ \mu & \mu & 1-\mu & 0 & 0 & 0\\ 0 & 0 & 0 & \frac{1}{2}-\mu & 0 & 0\\ 0 & 0 & 0 & 0 & \frac{1}{2}-\mu & 0\\ 0 & 0 & 0 & 0 & 0 & \frac{1}{2}-\mu \end{array}\right)\left\{\begin{array}{c} \varepsilon_{x}\\ \varepsilon_{\theta }\\ \varepsilon_{r}\\ \gamma_{\theta r}\\ \gamma_{xr}\\ \gamma_{x\theta } \end{array}\right\}$$

where $\sigma_{x}$, $\sigma_{\theta }$, and $\sigma_{r}$ are the normal stresses, $\tau_{\theta r}$, $\tau_{xr}$, and $\tau_{x\theta }$ are the shear stresses. *E* and *μ* are Young’s modulus and Poisson’s ratio, respectively.

The strain energy of the shell is:

(3) $$U=\frac{1}{2}\underset{\Omega }{∭}\left(\varepsilon_{x}\sigma_{x}+\varepsilon_{\theta }\sigma_{\theta }+\varepsilon_{r}\sigma_{r}+\gamma_{\theta r}\tau_{\theta r}+\gamma_{xr}\tau_{xr}+\gamma_{x\theta }\tau_{x\theta }\right)rd\theta drdx$$

The strain energy function of the shell expressed in terms of displacements can be obtained by substituting Equations (1) and (2) into Equation (3):

(4) $$U=\frac{E}{2(1+\mu )(1-2\mu )}\int_{0}^{L}\int_{R_{0}}^{R_{1}}\int_{0}^{2\pi }\left\{\begin{array}{c} \left(1-\mu \right)\left[{\left(\frac{\partial u}{\partial x}\right)}^{2}+{\left(\frac{\partial v}{r\partial \theta }+\frac{w}{r}\right)}^{2}+{\left(\frac{\partial w}{\partial r}\right)}^{2}\right]+\\ 2\mu \left(\frac{\partial v}{r\partial \theta }\frac{\partial u}{\partial x}+\frac{\partial u}{\partial x}\frac{w}{r}+\frac{\partial u}{\partial x}\frac{\partial w}{\partial r}+\frac{\partial v}{r\partial \theta }\frac{\partial w}{\partial r}+\frac{\partial w}{\partial r}\frac{w}{r}\right)+\\ \left(\frac{1}{2}-\mu \right)\left[{\left(\frac{\partial w}{r\partial \theta }+\frac{\partial v}{\partial r}-\frac{v}{r}\right)}^{2}+{\left(\frac{\partial u}{\partial r}+\frac{\partial w}{\partial x}\right)}^{2}+{\left(\frac{\partial u}{r\partial \theta }+\frac{\partial v}{\partial x}\right)}^{2}\right] \end{array}\right\}rd\theta drdx$$

The kinetic energy of cylindrical shells is expressed as:

(5) $$T=\frac{\rho }{2}\int_{0}^{L}\int_{R_{0}}^{R_{1}}\int_{0}^{2\pi }\left[{\left(\frac{\partial u}{\partial t}\right)}^{2}+{\left(\frac{\partial v}{\partial t}\right)}^{2}+{\left(\frac{\partial w}{\partial t}\right)}^{2}\right]rd\theta drdx$$

where *ρ* denotes the density of cylindrical shells.

The potential energy of the spring on the two boundary end surfaces of 3D cylindrical shells is

(6) $$U_{b}=\frac{1}{2}\int_{R_{0}}^{R_{1}}\int_{0}^{2\pi }\left[{\left(k_{u}^{0}u^{2}+k_{v}^{0}v^{2}+k_{w}^{0}w^{2}\right)}_{x=0}+{\left(k_{u}^{L}u^{2}+k_{v}^{L}v^{2}+k_{w}^{L}w^{2}\right)}_{x=L}\right]rd\theta dr$$

In this paper, to achieve the displacement continuity closure of the cylindrical shell, coupling springs are arranged on the two surfaces of *θ* = 0 and *θ* = 2π. The corresponding coupling potential energy is written as:

(7) $$U_{cp}=\frac{1}{2}\int_{R_{0}}^{R_{1}}\int_{0}^{L}\left[K_{u}{\left({\left.u\right|}_{\theta =0}-{\left.u\right|}_{\theta =2\pi }\right)}^{2}+K_{v}{\left({\left.v\right|}_{\theta =0}-{\left.v\right|}_{\theta =2\pi }\right)}^{2}+K_{w}{\left({\left.w\right|}_{\theta =0}-{\left.w\right|}_{\theta =2\pi }\right)}^{2}\right]rdxdr$$

### 2.3. The Unified Formulation with Chebyshev Discretization

In the present study, the CUF is used to construct the displacement fields of the 3D cylindrical shell. The displacements *u*, *v*, and *w* in the cylindrical coordinate system are:

(8a) $$u\left(x,\theta ,r\right)=\sum_{i=1}^{Z-1}u_{i}\left(x,\theta \right)F_{i}\left(r\right)=u_{0}\left(x,\theta \right)r^{0}+u_{1}\left(x,\theta \right)r^{1}+\cdots +u_{Z-1}\left(x,\theta \right)r^{Z-1}$$

(8b) $$v\left(x,\theta ,r\right)=\sum_{i=1}^{Z-1}v_{i}\left(x,\theta \right)F_{i}\left(r\right)=v_{0}\left(x,\theta \right)r^{0}+v_{1}\left(x,\theta \right)r^{1}+\cdots +v_{Z-1}\left(x,\theta \right)r^{Z-1}$$

(8c) $$w\left(x,\theta ,r\right)=\sum_{i=1}^{Z-1}w_{i}\left(x,\theta \right)F_{i}\left(r\right)=w_{0}\left(x,\theta \right)r^{0}+w_{1}\left(x,\theta \right)r^{1}+\cdots +w_{Z-1}\left(x,\theta \right)r^{Z-1}$$

where *F_i_* is Taylor-like expansions, and *Z* denotes the number of terms of the expansion. *u_i_*, *v_i_*, and *w_i_* denote the displacement variables, which are approximated by Chebyshev polynomials of the first kind.

(9a) $$u_{i}\left(x,\theta \right)=\sum_{m=0}^{M-1}\sum_{n=0}^{N-1}A_{imn}\cos\left[m\cos^{-1}\left(x\right)\right]\cos\left[n\cos^{-1}\left(\theta \right)\right]$$

(9b) $$v_{i}\left(x,\theta \right)=\sum_{m=0}^{M-1}\sum_{n=0}^{N-1}B_{imn}\cos\left[m\cos^{-1}\left(x\right)\right]\cos\left[n\cos^{-1}\left(\theta \right)\right]$$

(9c) $$w_{i}\left(x,\theta \right)=\sum_{m=0}^{M-1}\sum_{n=0}^{N-1}C_{imn}\cos\left[m\cos^{-1}\left(x\right)\right]\cos\left[n\cos^{-1}\left(\theta \right)\right]$$

in which *M* and *N* are the number of terms of Chebyshev polynomials. *A_imn_*, *B_imn_*, and *C_imn_* represent the unknown coefficients of the expansion.

Considering that the radial direction of the cylindrical shell structure, also known as the thickness direction, has smaller dimensions than the other two directions, the Taylor series expansion is adopted for the radial direction in this paper. The advantage of doing so is obvious, which can significantly reduce the dimensionality of the displacement expansion. On the other hand, the use of CUF can also effectively avoid Poisson’s locking and does not require correction of the material stiffness coefficients.

It should be noted that the domain of definition of the radial direction of the cylindrical shell in this paper needs to be transformed from $r\in \left[R_{0},R_{1}\right]$ to $\bar{r}\in \left[0,h\right]$ (*h* = *R*_1_ − *R*_0_). Thus, the Taylor-like expansions can be rewritten as $F_{i}\left(\bar{r}\right)$, and the energy expressions of the cylindrical shell then get rewritten as:

(10) $$\begin{array}{cc} & U=\frac{E}{2(1+\mu )(1-2\mu )}\int_{0}^{L}\int_{0}^{h}\int_{0}^{2\pi }\left\{\begin{array}{c} (1-\mu )\left[\begin{array}{c} {\left(\frac{\partial u}{\partial x}\right)}^{2}+{\left(\frac{\partial w}{\partial \bar{r}}\right)}^{2}+\\ {\left(\frac{\partial v}{\bar{r}+R_{0}\partial \theta }+\frac{w}{\bar{r}+R_{0}}\right)}^{2} \end{array}\right]+\;\\ 2\mu \left(\begin{array}{l} \frac{\partial v}{\left(\bar{r}+R_{0}\right)\partial \theta }\frac{\partial u}{\partial x}+\frac{\partial u}{\partial x}\frac{w}{\left(\bar{r}+R_{0}\right)}+\\ \frac{\partial u}{\partial x}\frac{\partial w}{\partial \bar{r}}+\frac{\partial v}{\left(\bar{r}+R_{0}\right)\partial \theta }\frac{\partial w}{\partial \bar{r}}+\frac{\partial w}{\partial \bar{r}}\frac{w}{\left(\bar{r}+R_{0}\right)} \end{array}\right)+\\ (\frac{1}{2}-\mu )\begin{array}{l} \left[\begin{array}{c} {\left(\frac{\partial w}{\left(\bar{r}+R_{0}\right)\partial \theta }+\frac{\partial v}{\partial \bar{r}}-\frac{v}{\left(\bar{r}+R_{0}\right)}\right)}^{2}+\\ {\left(\frac{\partial u}{\partial \bar{r}}+\frac{\partial w}{\partial x}\right)}^{2}+{\left(\frac{\partial u}{\left(\bar{r}+R_{0}\right)\partial \theta }+\frac{\partial v}{\partial x}\right)}^{2} \end{array}\right] \end{array} \end{array}\right\}\left(\bar{r}+R_{0}\right)d\theta d\bar{r}dx \end{array}$$

(11) $$T=\frac{\rho }{2}\int_{0}^{L}\int_{0}^{h}\int_{0}^{2\pi }\left[{\left(\frac{\partial u}{\partial t}\right)}^{2}+{\left(\frac{\partial v}{\partial t}\right)}^{2}+{\left(\frac{\partial w}{\partial t}\right)}^{2}\right]\left(\bar{r}+R_{0}\right)d\theta d\bar{r}dx$$

(12) $$U_{b}=\frac{1}{2}\int_{0}^{h}\int_{0}^{2\pi }\left[{\left(k_{u}^{0}u^{2}+k_{v}^{0}v^{2}+k_{w}^{0}w^{2}\right)}_{x=0}+{\left(k_{u}^{L}u^{2}+k_{v}^{L}v^{2}+k_{w}^{L}w^{2}\right)}_{x=L}\right]\left(\bar{r}+R_{0}\right)d\theta d\bar{r}$$

(13) $$U_{cp}=\frac{1}{2}\int_{0}^{h}\int_{0}^{L}\left[K_{u}{\left({\left.u\right|}_{\theta =0}-{\left.u\right|}_{\theta =2\pi }\right)}^{2}+K_{v}{\left({\left.v\right|}_{\theta =0}-{\left.v\right|}_{\theta =2\pi }\right)}^{2}+K_{w}{\left({\left.w\right|}_{\theta =0}-{\left.w\right|}_{\theta =2\pi }\right)}^{2}\right]\left(\bar{r}+R_{0}\right)dxd\bar{r}$$

### 2.4. Solving Procedures

The Lagrange function of the cylindrical shell with elastic boundaries can be written as:

(14) $$L=T-\left(U+U_{cp}+U_{b}\right)$$

Substituting Equation (8) into Equation (14), and making the partial derivatives of the Lagrangian energy function regarding the unknown coefficients equal to zero:

(15) $$\begin{array}{cc} \frac{\partial L}{\partial \alpha }=0 & \alpha =A_{imn},B_{imn},C_{imn} \end{array}$$

The following system characteristic equations can be summed up in matrix form:

(16) $$\left(M\omega^{2}-K\right)q=0$$

where M and K represent the mass matrix and stiffness matrix of 3D cylindrical shells, respectively. $q={\left[\begin{array}{ccc} A_{imn} & B_{imn} & C_{imn} \end{array}\right]}^{T}$ is the vector of unknown coefficients. The detailed expressions of the mass and stiffness matrix are

(17) $$M=\left[\begin{array}{ccc} M_{uu} & M_{uv} & M_{uw}\\ M_{vu} & M_{vv} & M_{vw}\\ M_{wu} & M_{wv} & M_{ww} \end{array}\right]$$

(18) $$K=\left[\begin{array}{ccc} K_{uu} & K_{uv} & K_{uw}\\ K_{vu} & K_{vv} & K_{vw}\\ K_{wu} & K_{wv} & K_{ww} \end{array}\right]$$

where the dimensions of the submatrix **M***_ij_* and **K***_ij_* (*i*, *j* = *u*, *v*, *w*) are *M* × *N* × *Z.* By using the eigenvalue function ‘*eigs*’ in MATLAB, Equation (16) can be solved quickly to obtain the frequency and model characteristics of the shells. The detailed expressions of stiffness matrix **K** and mass matrix **M** can be found in Appendix A.

## 3. Results and Discussion

In this section, the free vibration characteristics of 3D cylindrical shells are investigated. Based on the theoretical formulations presented in the preceding section, numerous vibration results for cylindrical shells with different boundary conditions are presented, which prove the correctness, efficiency, and reliability of the solution proposed in this paper. Firstly, we will determine the stiffness values of the coupling spring and the boundary spring in the present method for the next calculation, then the convergence and efficiency of the present scheme are studied. Next, some computational results of the current approach are given and the accuracy of the model in this paper is checked by comparing it with the results in the open literature, including the modal of shells with various boundary conditions and their combinations. Finally, parameter studies for cylindrical shells with different geometric features are investigated.

### 3.1. Spring Parameters and Convergence Study

In this paper, the general artificial boundary spring technology is implemented to simulate the boundary forces and displacements of each end of the shells. Therefore, by setting different stiffness parameters for three groups of boundary surface springs, arbitrary boundary conditions of the 3D cylindrical shell can be obtained. In the current study, a clamped end of the shell can be sassily achieved by setting the stiffness of all boundary surface springs to a large value of 10^14^, while the free boundary conditions are achieved by setting the stiffness value to 0. The symbols C, S, F, and E1 are used to represent the clamped, simply supported, free, and elastically boundary conditions, respectively. The corresponding spring parameters are displayed in Table 2. Thus, the boundary conditions at both ends of the shells can be represented by two letters. For example, CF means that the 3D shell is fully constrained at surface *x* = 0 and free at surface *x* = *L*.

The CUF utilized in the current solution holds infinite expansion potential in theory. Nevertheless, truncation becomes necessary in practical calculations to satisfy the computational efficiency requirements. Subsequently, the task at hand involves identifying the suitable truncation numbers *M*, *N*, and *Z* for the displacement expansions in the axial, circumferential, and radial directions, respectively. Table 3 outlines the investigation of the first six non-dimensional frequencies of a 3D cylindrical shell with free edges, considering various truncation numbers. The results provide valuable insights into the optimal truncation numbers required for accurate and efficient calculations of the vibrational behavior of cylindrical shells. The dimensionless frequency parameter is set as $\Omega =\omega R_{1}\sqrt{\rho /E/\left(2+2\mu \right)}$. The geometric and material constants of the shell in Table 3 are inner radius *R*_0_ =1, outer radius *R*_1_ = 1.5, length *L* = 4, and Poisson’s ratio *μ* = 0.3. This paper, in particular, does not take into account the rigid body modes at completely free boundaries. Nonetheless, the results showcased in Table 3 demonstrate the excellent convergence of the current method, with the dimensionless frequency ultimately converging to a constant value when the dimensions of *M* and *N* surpass 12. Additionally, when both *M* and *N* are set to 18, the maximum disparity observed between the axial truncation number *Z* taken as 3 and 4 is merely 0.0573%, thereby affirming the robustness and reliability of the proposed approach. Table 4 lists the natural frequencies of a cylindrical shell with CF boundaries. The geometric parameters of the shell are set as *R*_0_ = 0.015, *R*_1_ = 0.03, and *L* = 2. As demonstrated in Table 4, it can be observed that with the increase in truncation numbers *M* and *N*, there is a corresponding decrease in the error between their natural frequency results. Furthermore, this error does not exceed 0.02%. It is important to note that due to the relatively thin thickness of the cylindrical shell under consideration, the axial truncation number *Z* has a negligible effect on the results. In light of these findings, the truncation numbers for the present work have been uniformly selected as *M* = *N* = 16 and *Z* = 4 for all subsequent calculations.

### 3.2. Results Validation

In this section, the accuracy and generality of the proposed solution are rigorously evaluated through a comparative analysis of the calculation results presented in this paper with those obtained from other relevant literature and finite element analyses. The results of this comparison serve to confirm the validity and robustness of the proposed solution. Table 5 presents the non-dimensional frequency parameters $\Omega =\omega R_{1}\sqrt{\rho /E}$ for an FF solid cylinder, which are compared with the results from existing literature. The geometric parameter of the cylinder is *L*/*R*_1_ = 4. As evidenced by the data presented in the table, it can be observed that there is a high degree of consistency between the current results and the reference data. It is important to note that the reference literature employed the Rayleigh—Ritz method, wherein the cylindrical circumferential displacements were characterized in terms of circumferential wave numbers, and the frequency does not increase with wave numbers.

**Figure 3:**
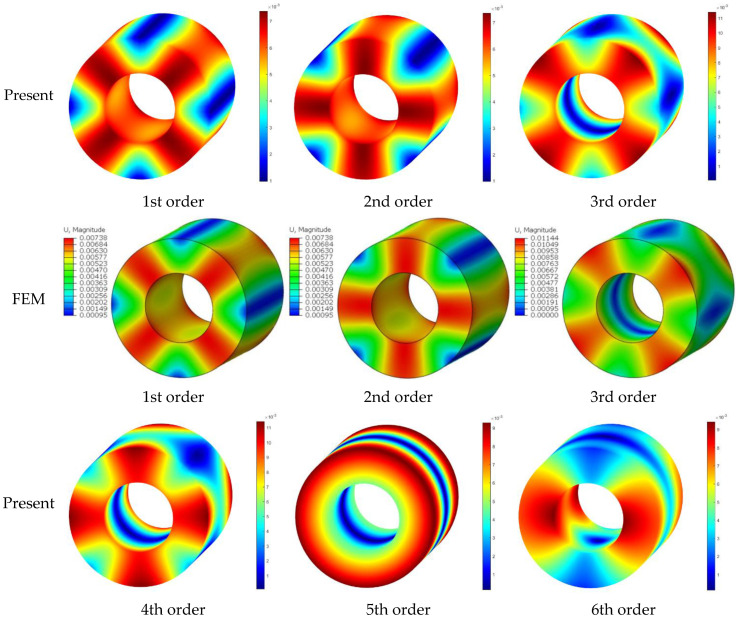

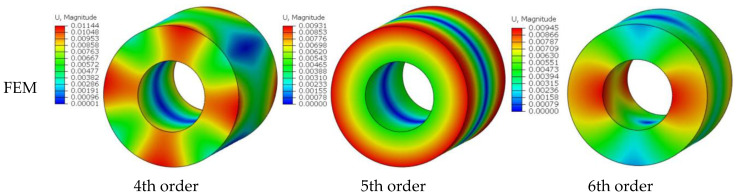
The comparison of the first 6 mode shapes for an FF cylindrical shell between the present method and FEM.

Table 6 shows the dimensionless frequencies $\Omega =\omega R_{1}\sqrt{\rho /E/\left(2+2\mu \right)}$ determined by the various methods in order to further contrast the differences between the approach used in this paper and the modeling approach that takes the cylindrical shell’s circumferential symmetry into account. The geometric and material parameters of the shell are: inner–outer ratio *R*_0_/*R*_1_ = 0.5, length–radius ratio *L*/*R*_1_ = 2, and *μ* = 0.3. The total number of meshes for the FEM model is 124,000, and the mesh type is a 3D hexahedral mesh. As indicated by the data presented in the table, it can be observed that the current method yields highly accurate calculation results when compared with the literature method. Furthermore, the frequencies of the cylindrical shell can be determined in a sequential manner. When compared with the calculation results obtained from FEM, it is evident that the method proposed in this paper exhibits a high degree of consistency. In particular, the first twenty orders of modalities can be accurately calculated using this method. The corresponding mode shapes are drawn in Figure 3. From the comparison of modal pictures, it can be seen that the current solution conforms well with the FEM, and all the vibration patterns can be effectively represented. It should be particularly noted that the fifth-order mode is an asymmetric mode.

Table 7 presents the frequency parameters $\Omega =\omega h/\pi \sqrt{\rho /E/\left(2+2\mu \right)}$ for the cylindrical shell under SS boundary conditions. Three length—radius ratios *L*/*R* are listed in the table, and the geometric parameters of the shell are *h*/*R* = 0.1, in which *R* indicates the radius of the middle surface of the shell. Based on the results presented, it can be concluded that there is a high degree of agreement between the current results and those reported in the reference work. It is hypothesized that the primary source of error between the two sets of results may be attributed to the differences in the displacement admissible functions selected by the respective methods.

**Figure 4:**
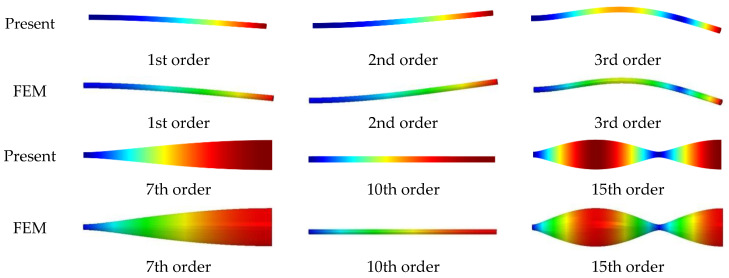
The modal digraph of a CF cylindrical shell.

Next, we study cylindrical shells with much larger axial dimensions than radial dimensions; typical structures of this type include pipelines, artillery, rockets, etc. Table 8 lists the first 8 frequency parameters $\Omega =\omega L\sqrt{\rho /E/(2+2\mu )}$ for a cylindrical shell with various boundary conditions, the results in the table are obtained by the present method and FEM. The geometric parameters of the shell are the same as the shells in Table 4. From the results of this table, the precision of the current method is demonstrated once more. The maximum difference of the calculation of the method in this paper does not exceed 1.163% compared with the results of FEM calculation. The primary reason for the observed differences between the results obtained using the current method and those obtained using finite element analyses can be attributed to the manner in which the elastic boundary is imposed. In the case of FEM, the elastic boundary is imposed through discrete mesh nodes. In contrast, the current method directly imposes the elastic boundary on the entire boundary surface. The results of this comparison serve to demonstrate the effectiveness of the current method in accurately modeling the elastic boundary. Figure 4 presents some modal diagraphs of the cylindrical shell with CF boundary conditions in Table 8. The mode shapes of this type of cylindrical shell are similar to that of beam structures, which can be seen from the first 3rd-order modal diagram. Meanwhile, the current method can accurately simulate the radial asymmetric mode of cylindrical shells. The accuracy of the present solution is again demonstrated by the modal comparison.

### 3.3. Numerical Study

Following the successful verification of the accuracy of the current method in modeling the free vibration of 3D cylindrical shells under a range of boundary conditions and geometric parameters, this subsection will present new vibration results obtained using this method for 3D cylindrical shells. The first 9 dimensionless frequency parameters $\Omega =\omega R_{1}\sqrt{\rho /E/(2+2\mu )}$ of the cylindrical shell with different boundary conditions and lengths are listed in Table 9. The material and geometric parameters of the shell in this table are set as *E* = 206 GPa, *μ* = 0.3, *ρ* = 7850 kg/m^3^, *R*_0_ = 0.1, and *R*_1_ = 0.3. The data presented in the table reveal a decreasing trend in the frequency parameter of the cylindrical shell as the length is increased. This phenomenon can be attributed to the decrease in stiffness that is observed in longer shells, which renders them more susceptible to vibrations. Furthermore, variations in the boundary conditions and length of the shell tend to alter the manifestation of the shell’s symmetric modes, thereby affecting its vibrational characteristics.

Table 10 presents the first 8 dimensionless frequency parameters $\Omega =\omega L\sqrt{\rho /E/(2+2\mu )}$ of the cylindrical shell with different boundary conditions and radius of the middle surface. The material and geometric parameters of the shell in this table are set as *E* = 206 GPa, *μ* = 0.3, *ρ* = 7850 kg/m^3^, *h* = 0.1, *R*_0_ = *R* − h/2, *R*_1_ = *R* + *h*/2, and *L* = 2. As indicated by the data presented, it can be observed that the frequency parameter of the shell exhibits a decreasing trend with increasing radius. This phenomenon can be attributed to the fact that, as the radius of the shell increases, its mass also increases. It is important to note that changes in the radius of the cylindrical shell do not appear to have a significant impact on the distribution of its symmetric modes. Furthermore, as evidenced by the data presented in Table 9 and Table 10, it can be seen that the frequency results obtained for both elastic and free boundaries are largely consistent. This suggests that the stiffness of the springs has not had a significant impact on the structure.

**Figure 5:**
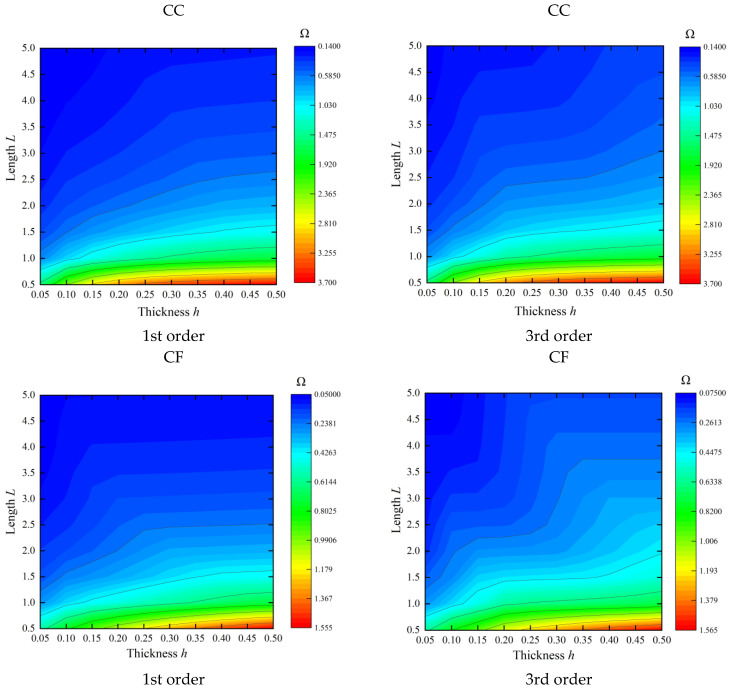
The variations of the frequency parameters $\Omega =\omega \sqrt{\rho /E}$ for cylindrical shells versus varying thickness and length.

Next, we will conduct a more detailed investigation into the effects of both thickness and length on the frequency characteristics of the cylindrical shell. Figure 5 shows the changes in the dimensionless frequency parameters $\Omega =\omega \sqrt{\rho /E}$ of the cylindrical shell with different thicknesses *h* and lengths *L*. The material and geometric parameters of the shell in this table are set as *E* = 206 GPa, *μ* = 0.3, *ρ* = 7850 kg/m^3^, *R* = 1, *R*_0_ = *R* − *h*/2, and *R*_1_ = *R* + *h*/2. As depicted in Figure 5, it can be observed that the frequency of the cylindrical shell structure exhibits an increasing trend with increasing thickness and a decreasing trend with increasing length. Furthermore, it is important to note that, as the length of the shell increases, the rate at which its frequency decreases with increasing thickness becomes slower.

## 4. Conclusions

The 3D vibration characteristics of the cylindrical shell with generally restrained ends are studied in this paper. A new 3D vibration analysis model applicable to the cylindrical shell is established by combining the CUF and Chebyshev polynomials. The elastic constraint boundaries at both ends of the cylindrical shell were simulated using the penalty function technique during the modeling process. The frequency and modal properties of cylindrical shells with various boundary conditions and geometrical parameters are calculated and compared to results from related literature and FEM. The conclusions of the present study can be drawn as follows:

(1) The CUF model used in this paper is extended with Chebyshev polynomials and Taylor series for displacements in the axial, circumferential, and radial directions of the cylindrical shell, which effectively reduces the 3D model’s dimensionality.
(2) The model presented in this study can be used to investigate the free vibration features of elastically constrained, 3D cylindrical shells. The model has good convergence, according to the results of the pertinent calculations. Additionally, the model’s great computational correctness and universality can be demonstrated by comparison with relevant literature and finite element results.
(3) The frequency parameter of the shell decreases with increasing length, since the longer the shell, the lower the stiffness, thus the higher the proneness to vibration. The frequency parameter of the shell decreases with increasing radius, mainly because the mass of the shell increases with increasing radius. The frequency of the cylindrical shell structure increases with thickness, and the longer the length, the slower the decreasing trend of its frequency with the increase of the thickness of shells.
(4) The main drawback of the study in this paper is the inability to analyze the cylindrical shell structure with variable thickness characteristics, and the strong nonlinear characteristics are not considered.
(5) The research in this paper can provide support for the optimal design of cylindrical shell-like gun tube structures and dynamics performance prediction in the future, and can be further extended to the vibration characteristics analysis of composite cylindrical shells.

## Figures and Tables

**Table 1:** Recent studies on the vibration of cylindrical shell structures.

Authors	Materials	Elastic Theory	Method
Xiang et al. [11]	Laminated composites	First-order shear deformation theory	Meshless global collocation method
Tornabene et al. [12]		Higher-order shear deformation theory	Generalized Differential Quadrature method
Wang et al. [13]	Functionally graded sandwich materials	First-order shear deformation theory	Fourier series expression method
Fan et al. [14]	Functionally graded materials		Walsh series method
Chen et al. [15]	Functionally graded graphene platelets-reinforced materials		Chebyshev–Lagrangian approach
Liu et al. [16]	Functionally graded materials		Wave-based method
Guo et al. [17]	Laminated composites		Spectral-Tchebychev technique
Qin et al. [18]	Isotropic materials	Sanders shell theory	Rayleigh—Ritz method
Rahimi et al. [19]	Graphene platelet-reinforced porous composites	3D elastic theory	Differential quadrature method
Zheng et al. [20]	Isotropic materials	Donnell—Mushtari shell theory	Modified Fourier series method

**Table 2:** Spring parameters for different boundary restraints.

Boundary Conditions	Spring Parameters		
	*k_u_*	*k_v_*	*k_w_*
C	10^14^	10^14^	10^14^
S	0	10^14^	10^14^
F	0	0	0
E1	10^4^	10^4^	10^4^

**Table 3:** The convergence of the first 6 dimensionless frequency parameters $\Omega =\omega R_{1}\sqrt{\rho /E/\left(2+2\mu \right)}$ for the FF cylindrical shell.

*Z*	*M* × *N*	Modes					
		1	2	3	4	5	6
3	8 × 8	0.6582	0.6858	0.7163	0.7207	1.1780	1.2040
	10 × 10	0.5814	0.5828	0.6440	0.6445	1.1781	1.1787
	12 × 12	0.5782	0.5782	0.6415	0.6416	1.1781	1.1789
	14 × 14	0.5782	0.5782	0.6416	0.6417	1.1781	1.1792
	16 × 16	0.5782	0.5782	0.6417	0.6417	1.1781	1.1792
	18 × 18	0.5782	0.5782	0.6417	0.6417	1.1781	1.1792
4	8 × 8	0.6576	0.6855	0.7158	0.7203	1.1780	1.2036
	10 × 10	0.5810	0.5824	0.6437	0.6441	1.1781	1.1783
	12 × 12	0.5779	0.5779	0.6412	0.6412	1.1781	1.1785
	14 × 14	0.5779	0.5779	0.6413	0.6413	1.1781	1.1788
	16 × 16	0.5779	0.5779	0.6414	0.6414	1.1781	1.1788
	18 × 18	0.5779	0.5779	0.6414	0.6414	1.1781	1.1788

**Table 4:** The convergence of the first 6 natural frequencies for the CF cylindrical shell.

*Z*	*M* × *N*	Modes					
		1	2	3	4	5	6
3	8 × 8	66.284	99.632	141.104	159.373	218.621	251.441
	10 × 10	12.368	13.422	75.151	75.310	208.602	208.671
	12 × 12	12.033	12.034	75.022	75.035	208.373	208.474
	14 × 14	12.027	12.027	74.989	75.002	208.291	208.381
	16 × 16	12.024	12.024	74.970	74.982	208.247	208.327
	18 × 18	12.022	12.022	74.960	74.971	208.226	208.297
4	8 × 8	66.110	99.516	140.831	159.131	218.567	251.287
	10 × 10	12.365	13.415	75.150	75.308	208.599	208.668
	12 × 12	12.033	12.034	75.021	75.035	208.370	208.472
	14 × 14	12.027	12.027	74.988	75.001	208.288	208.378
	16 × 16	12.024	12.024	74.969	74.981	208.244	208.324
	18 × 18	12.022	12.022	74.959	74.970	208.223	208.295

**Table 5:** Comparison of the first 9 frequency parameters $\Omega =\omega R_{1}\sqrt{\rho /E}$ for an FF solid cylinder.

Method	Mode Number								
	1	2	3	4	5	6	7	8	9
Liew [3]	0.9594	0.9594	0.9742	1.5467	1.7751	1.7751	1.9483	2.5938	2.5938
Ye [9]	0.9595	0.9595	0.9742	1.5467	1.7751	1.7751	1.9483	2.5938	2.5938
Present	0.9595	0.9595	0.9742	1.5467	1.7751	1.7751	1.9483	2.5939	2.5939
Diff L	0.01%	0.01%	0	0	0	0	0	0.004%	0.004%
Diff Y	0	0	0	0	0	0	0	0.004%	0.004%

**Table 6:** Comparison of the frequency parameters $\Omega =\omega R_{1}\sqrt{\rho /E/(2+2\mu )}$ for an FF cylindrical shell.

Mode Number	Method			
	Present	FEM	Ye [9]	Zhou [5]
1	0.9703	0.9686	0.9700	0.9700
2	0.9703	0.9686		
3	1.0454	1.0429	1.0451	1.0451
4	1.0454	1.0429		
5	1.5708	1.5695		
6	1.6041	1.6036	1.6041	1.6041
7	1.6041	1.6036		
8	1.8932	1.8891	1.8932	1.8932
9	1.8932	1.8891		
10	1.9354	1.9288	1.9351	1.9351
11	1.9354	1.9288		
12	2.0424	2.0413		
13	2.1513	2.1489		
14	2.2883	2.2834		
15	2.2885	2.2835		
16	2.3047	2.2969		
17	2.3488	2.3421		
18	2.3490	2.3421		
19	2.4807	2.4764	2.4807	2.4807
20	2.4807	2.4764		

**Table 7:** Comparison of the frequency parameters $\Omega =\omega h/\pi \sqrt{\rho /E/(2+2\mu )}$ for the cylinder shell with SS boundaries.

*L*/*R*	Method	Mode Number							
		1	2	3	4	5	6	7	8
2	Ye [9]	0.01814		0.01906				0.03100	
	Ref. [33]	0.01814		0.01907				0.03100	
	Ref. [7]	0.01814		0.01906				0.03100	
	Present	0.01814	0.01814	0.01907	0.01907	0.02627	0.02640	0.03100	0.03100
1	Ye [9]			0.03642		0.03971			
	Ref. [33]			0.03643		0.03927			
	Ref. [7]			0.03641		0.03969			
	Present	0.03184	0.03184	0.03643	0.03644	0.03972	0.03972	0.04036	0.04066
0.5	Ye [9]						0.07616		0.07681
	Ref. [33]						0.07618		0.07684
	Ref. [7]						0.07607		0.07675
	Present	0.03184	0.03184	0.06368	0.06368	0.07614	0.07617	0.07617	0.07684

**Table 8:** Comparison of the first 8 frequency parameters $\Omega =\omega L\sqrt{\rho /E/(2+2\mu )}$ for the cylindrical shell with different boundary conditions.

B.C.	Method	Mode Number							
		1	2	3	4	5	6	7	8
FF	FEM	0.2987	0.2992	0.8180	0.8193	1.5883	1.5906	2.5927	2.5964
	Present	0.3015	0.3015	0.8253	0.8255	1.6021	1.6027	2.6144	2.6160
	Error	0.908%	0.761%	0.894%	0.761%	0.870%	0.760%	0.837%	0.758%
CF	FEM	0.0471	0.0472	0.2938	0.2943	0.8164	0.8177	1.5521	1.5825
	Present	0.0476	0.0476	0.2965	0.2966	0.8237	0.8240	1.5599	1.5959
	Error	0.940%	0.780%	0.917%	0.781%	0.889%	0.776%	0.505%	0.849%
CC	FEM	0.2981	0.2986	0.8142	0.8154	1.5768	1.5791	2.5680	2.5715
	Present	0.3009	0.3010	0.8215	0.8219	1.5900	1.5914	2.5882	2.5916
	Error	0.923%	0.796%	0.894%	0.800%	0.834%	0.781%	0.788%	0.782%
E1E1	FEM	0.2990	0.2994	0.8183	0.8195	1.5885	1.5908	2.5930	2.5966
	Present	0.3015	0.3015	0.8253	0.8255	1.6019	1.6027	2.6138	2.6160
	Error	0.850%	0.699%	0.861%	0.734%	0.842%	0.745%	0.805%	0.749%
CE1	FEM	0.0472	0.0473	0.2940	0.2944	0.8166	0.8178	1.5521	1.5826
	Present	0.0477	0.0477	0.2966	0.2966	0.8237	0.8240	1.5599	1.5959
	Error	1.163%	1.004%	0.886%	0.747%	0.875%	0.763%	0.505%	0.841%

**Table 9:** The first 8 frequency parameters $\Omega =\omega R_{1}\sqrt{\rho /E/(2+2\mu )}$ for a cylindrical shell with various boundary conditions and lengths.

B.C.	*L*	Mode Number							
		1	2	3	4	5	6	7	8
FF	0.5	1.4111	1.4114	1.4471	1.4478	1.8848	1.9471	1.9475	2.3868
	1	0.9424	1.0116	1.0116	1.4502	1.4507	1.4547	1.4554	1.4753
	2	0.3468	0.3468	0.4712	0.7431	0.7431	0.7554	0.9424	1.1672
CF	0.5	0.6446	0.6447	0.9423	1.5273	1.5278	1.5369	1.6856	1.6858
	1	0.2224	0.2224	0.4712	0.7670	0.7943	0.7944	1.4135	1.4589
	2	0.0638	0.0638	0.2356	0.3080	0.3080	0.3821	0.6919	0.6920
CC	0.5	1.6850	1.6853	1.8844	2.2074	2.2077	3.0542	3.0805	3.1789
	1	0.7525	0.7526	0.9423	1.4839	1.4841	1.5372	1.6250	1.6256
	2	0.2965	0.2965	0.4712	0.6361	0.6362	0.7669	0.9424	1.0349
E1E1	0.5	1.4111	1.4114	1.4471	1.4478	1.8848	1.9471	1.9476	2.3868
	1	0.9424	1.0116	1.0116	1.4502	1.4507	1.4547	1.4554	1.4753
	2	0.3468	0.3468	0.4712	0.7431	0.7431	0.7554	0.9424	1.1672
CS	0.5	1.4839	1.4841	1.5373	1.8844	2.0442	2.0446	2.1701	2.1703
	1	0.6361	0.6362	0.7670	0.9423	1.4496	1.4498	1.5763	1.5769
	2	0.2309	0.2310	0.3821	0.4712	0.5895	0.5896	0.9424	0.9998
SS	0.5	1.4056	1.4058	1.5403	1.5407	1.8844	1.9310	1.9315	2.3953
	1	0.5356	0.5357	0.9423	1.4057	1.4059	1.4753	1.5380	1.5387
	2	0.1679	0.1679	0.4712	0.5356	0.5357	0.7554	0.9424	0.9636
SF	0.5	0.9423	1.4502	1.4507	1.4851	1.4854	2.1343	2.1345	2.1419
	1	0.4712	0.7431	0.7431	1.4135	1.4546	1.4552	1.4753	1.4814
	2	0.2356	0.2495	0.2495	0.6407	0.6407	0.7068	0.7554	1.0665

**Table 10:** The first 8 frequency parameters $\Omega =\omega L\sqrt{\rho /E/(2+2\mu )}$ for a cylindrical shell with various boundary conditions and radius of the middle surface.

B.C.	*R*	Mode Number							
		1	2	3	4	5	6	7	8
FF	0.5	1.0189	1.0190	1.1123	1.1123	2.2115	2.2115	2.7943	2.7945
	1	0.2577	0.2577	0.3409	0.3409	0.7227	0.7227	0.8544	0.8545
	2	0.0644	0.0644	0.1153	0.1154	0.1817	0.1817	0.2876	0.2876
CF	0.5	0.7455	0.7456	1.0968	1.0969	1.5707	1.9305	1.9305	2.4139
	1	0.5667	0.5668	0.8178	0.8179	0.9481	0.9481	1.4415	1.4482
	2	0.4558	0.4559	0.4891	0.4905	0.6044	0.6044	0.7467	0.7647
CC	0.5	1.7689	1.7689	2.1506	2.1507	3.1326	3.1328	3.1414	3.1955
	1	1.4034	1.4035	1.4648	1.4648	1.8130	1.8228	2.0447	2.0447
	2	1.0391	1.0407	1.0830	1.0832	1.1400	1.1932	1.2511	1.2512
E1E1	0.5	1.0189	1.0190	1.1123	1.1123	2.2115	2.2115	2.7943	2.7945
	1	0.2577	0.2577	0.3409	0.3409	0.7227	0.7228	0.8544	0.8545
	2	0.0644	0.0644	0.1154	0.1154	0.1817	0.1817	0.2876	0.2876
CS	0.5	1.5776	1.5777	1.9193	1.9194	2.5359	3.0256	3.0257	3.0658
	1	1.2647	1.2648	1.3200	1.3201	1.7241	1.7332	1.9743	1.9743
	2	0.9271	0.9288	0.9864	0.9866	1.0426	1.0946	1.1882	1.1882
SS	0.5	1.4071	1.4071	1.7505	1.7505	2.8449	2.8450	3.0125	3.0127
	1	1.1398	1.1398	1.1980	1.1980	1.6504	1.6585	1.9478	1.9478
	2	0.8332	0.8350	0.9115	0.9116	0.9633	1.0001	1.0001	1.0117
SF	0.5	1.0454	1.0455	1.5707	1.7566	1.7567	2.3758	2.3758	2.8304
	1	0.2843	0.2843	0.7610	0.7611	1.4231	1.4295	1.4784	1.4785
	2	0.0863	0.0863	0.2174	0.2174	0.3946	0.3971	0.7157	0.7304

## Data Availability

Data sharing not applicable.

## Appendix A. Detailed Expressions for the Mass Matrix M and Stiffness Matrix K

The detailed expressions of the mass matrix **M** and stiffness matrix **K** in Equations (17) and (18) are given as follows.

$${Μ}_{uu}={Μ}_{vv}={Μ}_{ww}=\rho \left[\left(G_{x}⊗G_{\theta }\right)⊗G_{r}\right]$$

$${Μ}_{uv}={Μ}_{vu}={Μ}_{uw}={Μ}_{wu}={Μ}_{vw}={Μ}_{wv}=0$$

$$\begin{array}{l} K_{uu}=\frac{E\left(1-\mu \right)}{\left(1+\mu \right)\left(1-2\mu \right)}\left[\left(G_{x}^{1}⊗G_{\theta }\right)⊗G_{r}\right]+\frac{E}{2\left(1+\mu \right)}\left[\left(G_{x}⊗G_{\theta }\right)⊗G_{r}^{1}\right]+\\ \frac{E}{2\left(1+\mu \right)}\left[\left(G_{x}⊗G_{\theta }^{1}\right)⊗G_{r}\right]+k_{u}^{0}\left[\left(G_{x=1}⊗G_{\theta }\right)⊗G_{r}\right]+k_{u}^{L}\left[\left(G_{x=L}⊗G_{\theta }\right)⊗G_{r}\right]\\ +K_{u}\left\{\left[\left(G_{x}⊗G_{\theta =0}\right)⊗G_{r}\right]+\left[\left(G_{x}⊗G_{\theta =2\pi }\right)⊗G_{r}\right]-2\left[\left(G_{x}⊗G_{\theta =0,\theta^{\prime }=2\pi }\right)⊗G_{r}\right]\right\} \end{array}$$

$$K_{uv}=K_{vu}=\frac{E\mu }{\left(1+\mu \right)\left(1-2\mu \right)}\left[\left(G_{x}^{11}⊗G_{\theta }^{11}\right)⊗G_{r}\right]+\frac{E}{2\left(1+\mu \right)}\left[\left(G_{\theta }^{11}⊗G_{x}^{11}\right)⊗G_{r}\right]$$

$$\begin{array}{l} K_{uw}=K_{wu}=\frac{E\mu }{\left(1+\mu \right)\left(1-2\mu \right)}\left[\left(G_{x}^{11}⊗G_{\theta }\right)⊗G_{r}\right]+\frac{E\mu }{\left(1+\mu \right)\left(1-2\mu \right)}\left[\left(G_{x}^{11}⊗G_{\theta }\right)⊗G_{r}^{11}\right]+\\ \frac{E}{2\left(1+\mu \right)}\left[\left(G_{x}^{11}⊗G_{\theta }\right)⊗G_{r}^{11}\right] \end{array}$$

$$\begin{array}{cc} & K_{vv}=\frac{E(1-\mu )}{\left(1+\mu )(1-2\mu \right)}\left[\left(G_{x}⊗G_{\theta }^{1}\right)⊗G_{r}\right]+\frac{E}{2(1+\mu )}\left[\left(G_{x}⊗G_{\theta }\right)⊗G_{r}\right]+\\ & \frac{E}{2(1+\mu )}\left[\left(G_{x}^{1}⊗G_{\theta }\right)⊗G_{r}\right]+\frac{E}{2(1+\mu )}\left[\left(G_{x}⊗G_{\theta }\right)⊗G_{r}^{1}\right]-\frac{E}{\left(1+\mu \right)}\left[\left(G_{x}⊗G_{\theta }\right)⊗G_{r}^{11}\right]\\ & +k_{v}^{0}\left[\left(G_{x=1}⊗G_{\theta }\right)⊗G_{r}\right]+k_{v}^{L}\left[\left(G_{x=L}⊗G_{\theta }\right)⊗G_{r}\right]+\\ & K_{v}\left\{\left[\left(G_{x}⊗G_{\theta =0}\right)⊗G_{r}\right]+\left[\left(G_{x}⊗G_{\theta =2\pi }\right)⊗G_{r}\right]-2\left[\left(G_{x}⊗G_{\theta =0,\theta^{\prime }=2\pi }\right)⊗G_{r}\right]\right\} \end{array}$$

$$\begin{array}{cc} & K_{wv}=K_{wv}=\frac{E(1-\mu )}{\left(1+\mu )(1-2\mu \right)}\left[\left(G_{x}⊗G_{\theta }^{11}\right)⊗G_{r}\right]+\frac{E\mu }{\left(1+\mu )(1-2\mu \right)}\left[\left(G_{x}⊗G_{\theta }^{11}\right)⊗G_{r}^{11}\right]+\\ & \frac{E}{2(1+\mu )}\left[\left(G_{x}⊗G_{\theta }^{11}\right)⊗G_{r}^{11}\right]-\frac{E}{2(1+\mu )}\left[\left(G_{x}⊗G_{\theta }^{11}\right)⊗G_{r}\right] \end{array}$$

$$\begin{array}{cc} & K_{ww}=\frac{E(1-\mu )}{\left(1+\mu )(1-2\mu \right)}\left[\left(G_{x}⊗G_{\theta }\right)⊗G_{r}\right]+2\frac{E\mu }{\left(1+\mu )(1-2\mu \right)}\left[\left(G_{x}⊗G_{\theta }\right)⊗G_{r}^{11}\right]+\\ & \frac{E(1-\mu )}{\left(1+\mu )(1-2\mu \right)}\left[\left(G_{x}⊗G_{\theta }\right)⊗G_{r}^{1}\right]+\frac{E}{2(1+\mu )}\left[\left(G_{x}⊗G_{\theta }^{1}\right)⊗G_{r}\right]+\frac{E}{\left(1+\mu \right)}\left[\left(G_{x}^{1}⊗G_{\theta }\right)⊗G_{r}\right]\\ & +k_{w}^{0}\left[\left(G_{x=1}⊗G_{\theta }\right)⊗G_{r}\right]+k_{w}^{L}\left[\left(G_{x=L}⊗G_{\theta }\right)⊗G_{r}\right]+\\ & K_{w}\left\{\left[\left(G_{x}⊗G_{\theta =0}\right)⊗G_{r}\right]+\left[\left(G_{x}⊗G_{\theta =2\pi }\right)⊗G_{r}\right]-2\left[\left(G_{x}⊗G_{\theta =0,\theta^{\prime }=2\pi }\right)⊗G_{r}\right]\right\} \end{array}$$

where

$$G_{x}=\int_{0}^{L}\sum_{m=1}^{M-1}\sum_{m^{\prime }=1}^{M^{\prime }-1}\cos\left[m\cos^{-1}\left(x\right)\right]\cos\left[m^{\prime }\cos^{-1}\left(x\right)\right]dx$$

$$G_{\theta }=\int_{0}^{2\pi }\sum_{n=1}^{N-1}\sum_{n^{\prime }=1}^{N^{\prime }-1}\cos\left[n\cos^{-1}\left(\theta \right)\right]\cos\left[n^{\prime }\cos^{-1}\left(\theta \right)\right]d\theta$$

$$G_{r}=\int_{0}^{h}\sum_{i=1}^{Z-1}\sum_{i^{\prime }=1}^{Z^{\prime }-1}{\bar{r}}^{i}{\bar{r}}^{i^{\prime }}\left(\bar{r}+R_{0}\right)d\bar{r}$$

$$G_{x}^{1}=\int_{0}^{L}\sum_{m=1}^{M-1}\sum_{m^{\prime }=1}^{M^{\prime }-1}\frac{d\cos\left[m\cos^{-1}\left(x\right)\right]}{dx}\frac{d\cos\left[m^{\prime }\cos^{-1}\left(x\right)\right]}{dx}dx$$

$$G_{\theta }^{1}=\int_{0}^{2\pi }\sum_{n=1}^{N-1}\sum_{n^{\prime }=1}^{N^{\prime }-1}\frac{d\cos\left[n\cos^{-1}\left(\theta \right)\right]}{d\theta }\frac{d\cos\left[n^{\prime }\cos^{-1}\left(\theta \right)\right]}{d\theta }d\theta$$

$$G_{r}^{1}=\int_{0}^{h}\sum_{i=1}^{Z-1}\sum_{i^{\prime }=1}^{Z^{\prime }-1}{\frac{d\bar{r}}{d\bar{r}}}^{i}\frac{d{\bar{r}}^{i^{\prime }}}{d\bar{r}}\left(\bar{r}+R_{0}\right)d\bar{r}$$

$$G_{x}^{11}=\int_{0}^{L}\sum_{m=1}^{M-1}\sum_{m^{\prime }=1}^{M^{\prime }-1}\frac{d\cos\left[m\cos^{-1}\left(x\right)\right]}{dx}\cos\left[m^{\prime }\cos^{-1}\left(x\right)\right]dx$$

$$G_{\theta }^{11}=\int_{0}^{2\pi }\sum_{n=1}^{N-1}\sum_{n^{\prime }=1}^{N^{\prime }-1}\frac{d\cos\left[n\cos^{-1}\left(\theta \right)\right]}{d\theta }\cos\left[n^{\prime }\cos^{-1}\left(\theta \right)\right]d\theta$$

$$G_{r}^{11}=\int_{0}^{h}\sum_{i=1}^{Z-1}\sum_{i^{\prime }=1}^{Z^{\prime }-1}{\frac{d\bar{r}}{d\bar{r}}}^{i}{\bar{r}}^{i^{\prime }}\left(\bar{r}+R_{0}\right)d\bar{r}$$
